# Targeted Strategies for Henipavirus Therapeutics

**DOI:** 10.2174/1874357900701010014

**Published:** 2007-09-28

**Authors:** Katharine N Bossart, John Bingham, Deborah Middleton

**Affiliations:** CSIRO Livestock Industries, Australian Animal Health Laboratory, Geelong, Victoria 3220, Australia

## Abstract

Hendra and Nipah viruses are related emergent paramyxoviruses that infect and cause disease in animals and humans. Disease manifests as a generalized vasculitis affecting multiple organs, but is the most severe in the respiratory and central nervous systems. The high case fatality and person-to-person transmission associated with the most recent NiV outbreaks, and the recent re-emergence of HeV, emphasize the importance and necessity of effective therapeutics for these novel agents. In recent years henipavirus research has revealed a more complete understanding of pathogenesis and, as a consequence, viable approaches towards vaccines and therapeutics have emerged. All strategies target early steps in viral replication including receptor binding and membrane fusion. Animal models have been developed, some of which may prove more valuable than others for evaluating the efficacy of therapeutic agents and regimes. Assessments of protective host immunity and drug pharmacokinetics will be crucial to the further advancement of therapeutic compounds.

## INTRODUCTION

Hendra virus (HeV) and Nipah virus (NiV) are closely related highly pathogenic paramyxoviruses that have emerged independently in the past 15 years and continue to emerge in new locations. Flying foxes in the genus *Pteropus* are considered to be the natural reservoir for both viruses as demonstrated by seroconversion and isolation of HeV- and NiV-like viruses from bat tissues and secretions [1, 2]. Additionally, extensive serological surveys have not demonstrated the presence of HeV- or NiV-specific antibodies in other species. Indeed the flying fox geographic range encompasses all locations where HeV and NiV have been found. Paramyxoviruses are large, enveloped, negative-sense ssRNA viruses, and include such well-known members as measles virus (MeV), simian virus 5 (SV5), and respiratory syncytial virus (RSV) [3]. It is a diverse virus family, with various members causing common upper and lower respiratory tract infections to less common manifestations of neurological disease. In contrast, NiV and HeV are distinguished from all other paramyxoviruses most notably by their broad species tropism and high case fatality, and they have been classified into the new *Henipavirus* genus within the family *Paramyxoviridae* [4]. HeV has appeared sporadically in Australia since 1994 where infection has been transmitted from horses to humans (reviewed in [5]). Presumably horses become infected through spillover events from flying foxes, although no virus has been isolated from flying foxes during outbreaks. In horses, the disease presented as a severe respiratory infection, while one of the two reported human mortalities had severe respiratory disease, and the other succumbed to encephalitis 13 months following the presumed time of exposure. Recent outbreaks where horse fatalities were documented include 1999, 2004 and 2006 and although no human mortalities have occurred, one mildly ill, seroconverting, human case has been reported [6-8]. NiV first appeared in peninsular Malaysia and Singapore in 1998-9 and the majority of infections occurred in pigs with subsequent transmission to humans (reviewed in [9, 10]). In pigs, infection was largely subclinical; however, where clinical disease was observed, it manifested as respiratory and encephalitic disease with low fatality ratios for respiratory cases. In contrast, humans developed severe febrile encephalitis with high case fatality and up to 25% of NiV cases also exhibited respiratory signs including non-productive cough. Interestingly, both relapsing and late-onset encephalitis syndromes with significant fatality (∼18%) have been recognized following either acute NiV encephalitic episodes or non-encephalitic/ asymptomatic infection. NiV has re-emerged numerous times since 1998: twice in 2001 in Bangladesh and West Bengal India, again in 2003 in Bangladesh, three times in Bangladesh in 2004 and 2005, and most recently in 2007 in Nadia, India [11-15]. Significant observations in all of the Bangladesh and Indian NiV outbreaks have included a higher incidence of acute respiratory distress syndrome in conjunction with encephalitis, epidemiological findings consistent with person-to-person transmission [16], and higher case fatality ratios (∼75%). Furthermore, no intermediate or amplification host has been identified and direct transmission of NiV from the reservoir host to humans has been suggested. In West Bengal in 2001 there was no concurrent illness in animals and in Bangladesh in 2004 the common epidemiological link among cases was drinking fresh date palm sap [12, 17]. In general, it is believed that date palm sap is regularly contaminated by flying foxes and their excretions.

NiV and HeV are classified as zoonotic biosafety level 4 (BSL-4) viruses and infectious virus can only be studied at a handful of laboratories worldwide. Both viruses have also been included among the various pathogenic agents of biodefense concern and each are classified as priority pathogens in category C by the Centers for Disease Control and Prevention (CDC) and the National Institute of Allergy and Infectious Diseases (NIAID). The category C agents include high emerging pathogens with the potential for causing morbidity and mortality with major economic and health impacts. Henipaviruses in particular, could be engineered for mass dissemination because of their availability from natural sources and their relative ease of propagation and dissemination. Currently there are no specific antiviral therapies or vaccines available for treating or preventing NiV or HeV infection resulting from a natural outbreak, laboratory accident or deliberate misuse.

## PATHOGENESIS IN HUMANS

There have been two fatal cases of HeV infection in humans, the first presented with severe respiratory disease and upon autopsy the patient’s lungs had gross lesions of congestion, hemorrhage and edema associated with chronic alveolitis and syncytial cell formation. The second fatal case presented thirteen months post-exposure and exhibited leptomeningitis with lymphocytes and plasma cells and foci of necrosis in various parts of the brain parenchyma (reviewed in [18]). Multinucleate endothelial cells were also present in the viscera as well as in the brain. During the first NiV outbreak in Malaysia encephalitis killed 105 of 265 infected individuals [19]. Immuno- and histological features were described as a systemic endothelial infection accompanied by vasculitis, thrombosis, ischaemia and necrosis [20]. These changes were especially noted in the central nervous system (CNS). Immunohistochemical analyses demonstrated the widespread presence of NiV antigens both in neurons and other parenchymal cells in necrotic foci in the CNS and in endothelial cells of affected blood vessels. Evidence of vasculitis and endothelial infection was also seen in most organs examined. Disseminated endothelial cell infection, vasculitis, thrombosis and CNS parenchymal cell infection all appear to play essential roles in the fatal outcome of human NiV infection [18, 20]. In the more recent NiV outbreaks in India and Bangladesh, similar clinical signs were noted. However, a higher incidence of acute respiratory distress syndrome in conjunction with encephalitis was also described [11, 12].

## VIRUS ENTRY INTO HOST CELLS

Paramyxoviruses contain two major membrane-anchored envelope glycoproteins that are required for infection of a receptive host cell. All members contain an attachment glycoprotein, which binds the host cell receptor, while the second is the fusion (F) glycoprotein, which mediates pH-independent membrane fusion between the virus and its host cell (reviewed in [3]). Following virus attachment to a permissive host cell, paramyxovirus mediated membrane fusion occurs at neutral pH resulting in delivery of the nucleocapsid into the cytoplasm of the host cell. Most paramyxovirus attachment glycoproteins possess hemagglutinin and neuraminidase activities (HN), which bind sialic acid moieties. Virion attachment most likely occurs through binding of multiple sialic acid-expressing proteins on the cell surface. The morbillivirus attachment glycoproteins (H) contain only hemagglutinin activity and do not bind sialic acid. Three morbilliviruses, measles virus, canine distemper virus and rinderpest virus, can employ cell-surface proteins as viral receptors [21-26]. HeV and NiV possess attachment glycoproteins (G) that lack hemagglutinin and neuraminidase activities and are the only other paramyxoviruses known to utilize host cell proteins that do not contain sialic acid as viral receptors. Specifically, ephrin-B2 ligand was identified as a receptor utilized by HeV and NiV for infection [27, 28] and subsequently, ephrin-B3 ligand was identified as an alternate receptor for NiV [29]. Ephrin molecules are members of a family of receptor tyrosine kinase ligands and are highly conserved across vertebrate species [30, 31]. Ephrin ligands and their receptors play important roles in regulation of tissue assembly and cell migration [30] and are critical to brain function, neuronal networking and morphogenesis [32]. Ephrin-B2 ligand is an artery-specific protein that mediates blood vessel development and maturation [33, 34]. Neurons smooth muscle, arterial endothelial cells and capillaries all exhibit high levels of ephrin-B2 ligand expression consistent with the known tissue tropism of HeV and NiV *in vivo*. Ephrin-B3 ligand is expressed in certain neural subsets and may play an important role in HeV and NiV infection in the CNS. All paramyxoviruses F glycoproteins, including HeV and NiV, are typical class I fusion proteins that utilize two internal heptad repeats to mediate membrane merger (reviewed in [35]). Because both attachment and fusion are critical steps for productive infection, molecules that interfere with either process represent potential antiviral agents and in fact the most extensively characterized novel therapeutics fall within this category as will be discussed below.

## ANIMAL MODELS

HeV and NiV are currently classified as BSL-4 agents and consequently human efficacy studies for testing potential therapeutic products are not easily achievable. In 2002, the U.S. Food and Drug Administration (FDA) implemented the Animal Efficacy Rule for the development of therapeutic products under these circumstances. Specifically, FDA can rely on evidence derived from animal studies in the evaluation of product effectiveness when particular criteria are met, such as a well-understood mechanism for both the pathogenicity of the agent and the underlying mode of action of the product. Importantly, the therapeutic effect must also be demonstrated in more than one animal species. Once these criteria have been met, human clinical trials could commence, most likely in populations at high risk of natural infection by HeV and NiV.

A strong epidemiological association existed in Malaysia between human NiV infection and close direct contact with pigs, especially sick and dying animals. No direct association with flying foxes was made. An epidemiological link has been noted between HeV infection in people and horses dying from HeV disease. Again, neither HeV disease nor seroconversion has ever been identified in wildlife carers who came into close and regular contact with sick and injured bats. While both horses and pigs have been experimentally infected with HeV and NiV, respectively [42, 44], neither represents a practical option for multiple experimental efficacy studies. Large animals such as horses are difficult to manage in sufficient numbers under BSL-4 conditions and in pigs NiV infection is mostly inconsequential from a clinical perspective. For the purposes of this review we will focus on the smaller animal model systems that have been explored (Table 1). There was no serological evidence of NiV infection in rodents in Malaysia [9, 45]. However, attempts were made to infect mice experimentally. NiV and HeV do not cause disease in mice after subcutaneous administration (5,000 TCID_50_ HeV) (Crameri, G and Eaton, B.T., unpublished observations) or with either an intranasal (6x10^5^ pfu NiV) or intraperitoneal (10^7^ pfu NiV) challenge of NiV [40], although the HeV is lethal if administered intracranially. Rabbits are not susceptible to HeV associated disease when challenged subcutaneously (5,000 TCID_50_ HeV) [46]. Guinea pigs were experimentally infected with HeV soon after its discovery (5,000-50,000 TCID_50_ HeV) [46] and developed generalized vasculitis affecting lung, kidney, spleen, lymph nodes, gastrointestinal tract, and skeletal and intercostal muscles [36, 38]. However, in spite of vascular involvement of the lung, there was little or no pulmonary edema. NiV infection in the guinea pig (50,000 TCID_50_ NiV) clinically manifested as ruffled fur and abnormal (less fearful) behavior [39] with gross pathology limited to edema of the mesentery, broad ligament, and retroperitoneal tissues. Significant microscopic pathology included vasculitis with fibrinoid necrosis and endothelial syncytial cell formation in the myocardium, kidney, lymph node, spleen, myometrium, retroperitoneal tissues, and submucosal vessels of the bladder. Oophoritis and the presence of hemorrhagic corpora lutea were also noted together with endometrial degeneration and necrosis accompanied by multinucleated cells.

A golden hamster animal model for acute NiV virus infection has been established where, importantly, encephalitis and neuron infection were demonstrated similar to those seen in NiV-infected humans [40]. The LD_50_ values for hamsters inoculated by intraperitoneal and intranasal routes were 270 pfu and 47,000 pfu, respectively. Notably, the brain was the most severely affected organ in terms of vascular and parenchymal lesions. Neurons in the vicinity of vascultis showed numerous cytoplasmic eosinophilic inclusion bodies and both viral antigen and RNA were found extensively throughout neurons. Ultrastructural analysis revealed cytoplasmic inclusions composed of defined herringbone nucleocapsids typical of paramyxoviruses. However, although HeV and NiV clearly cause systemic disease in humans, NiV was not detected in serum samples from infected hamsters. Moreover, the pathology seen in the lungs and kidneys of NiV-infected hamsters differed from that seen in HeV-infected horses and NiV-infected humans. HeV infection of hamsters has not been reported.

Experimental HeV infection of cats has been performed and findings from those studies were similar to those of horses and humans, namely generalized vascular disease with the most severe effects seen in the lung [36, 44]. NiV infection in cats was comparable to that observed with HeV except that with NiV there was also extensive inflammation of the upper and lower respiratory tract epithelium, associated with the presence of viral antigen [42], similar to the severe respiratory disease observed in humans in the recent NiV outbreaks in Bangladesh. Cats succumb to infection 6 to 10 days following parenteral inoculation of as low as 500 TCID_50_ NiV [43], or oronasal administration of 50,000, TCID_50_ of low passage, plaque purified HeV [41, 42, 46] or NiV (Bossart, K., Bingham, J. and Middleton, D., unpublished data). Gross pathology common to both HeV and NiV infection of cats consisted of hydrothorax, dense purple-red consolidation in the lung with fluid accumulation and froth in the bronchi (reviewed in [18]. Histologically cats infected with NiV develop a necrotizing alveolitis, with necrotic foci developing within a range of other organs, particularly kidney, spleen, lymph nodes, bladder, ovaries, adrenal and meninges. Studies on cats that were euthanized early in the course of the infection (1 day after the onset of fever, as determined by radiotelemetry measurement of body temperature) indicated that alveolar infection by NiV preceded vascular infection suggesting an increased tropism for alveolar epithelium as compared to vascular tissue (Bingham, J., unpublished observation). Thus evidence to date indicates that the cat represents an animal model in which henipavirus induced pathology closely resembles the lethal respiratory disease caused by HeV and NiV in humans.

Experimental NiV infection in Pteropid bats has been attempted [39]. All bats that were challenged with 50,000 TCID_50_ NiV remained clinically well throughout the study period and no febrile responses were recorded following NiV inoculation *via* a parenteral route. Challenged bats developed a sub-clinical infection characterized by episodic viral shedding in urine, limited presence of virus within selected viscera and seroconversion. No gross abnormalities were identified on post-mortem examination of animals at various times post exposure; all bat tissues were negative upon immunohistochemical labeling for NiV antigen.

**Fig. (1):**
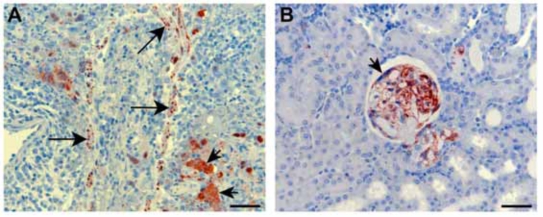
Histopathology and immunohistochemistry associated with NiV infection in ferrets. **A**: Severe necrotizing alveolitis and vasculitis with positive staining for NiV nucleoprotein antigen in blood vessel wall (long arrows) and syncytial cells (short arrows). **B**: Acute glomerular degeneration with associated NiV nucleoprotein antigen in ferret kidney. A syncytial cell (arrow) is visible along the inner membrane of Bowman's capsule. Immunohistochemistry, haematoxylin counterstain. Scale bar for all images = 50 µm.

Recently, we have been evaluating the potential of the ferret as an improved animal model for NiV infection. Ferrets have emerged as important animal models for several major respiratory diseases including highly pathogenic avian influenza [47], severe acute respiratory syndrome (SARS) [48], and also morbilliviruses [49], the closest relatives to HeV and NiV. Significant similarity exists between ferret and human lung physiology and morphology [50] and ferrets have been used previously for toxicology and biological safety assessment studies [51]. We have confirmed that ferrets are susceptible to NiV infection with disease developing 6 to 10 days following oronasal administration of 500 to 50,000 TCID_50_ (Bossart, K., Bingham, J. and Middleton, D., unpublished data). A lower dose of 50 TCID_50_ failed to produce infection as defined by fever, illness, detection of virus, viral antigen or viral genome, histopathological lesions or seroconversion. Infection first manifested as fever and this was followed by inappetance and severe depression. A mild increase in respiratory rate in some animals was attributable to fever with one ferret showing tremors and hindlimb weakness. Gross pathology included subcutaneous edema of the head, hemorrhagic lymphadenopathy of submandibular, retropharyngeal and sometimes visceral lymph nodes, numerous diffuse pin-point hemorrhagic nodules scattered throughout the pulmonary parenchyma and petechial hemorrhages of the renal cortex. Histopathological lesions included focal necrotizing alveolitis, vasculitis, degeneration of glomerular tufts, and focal necrosis in a range of other tissues, including lymph nodes, spleen, adrenal cortex, bladder and ovary. Lesions in each case were associated with significant quantities of viral antigen, as determined by immunohistochemical staining (Fig. 1). Syncytial cells were also frequently present in lesions. NiV genome was detected in blood, brain, liver, testes, adrenal, kidney, lung, lymph node and spleen. These preliminary results are encouraging and indicate that the ferret may be another suitable model for human infection.

The golden hamster, cat and ferret are practical laboratory animal models that can be used to evaluate aspects of disease caused by either HeV or NiV, and each is more amenable to therapeutic efficacy testing than large domestic animals particularly for these BSL-4 pathogens. In hamsters NiV-associated encephalitis was unique among the small animal models; however, it is unclear if this was due to the length of clinical course, where animals were kept until death occurred naturally [40]. In all other studies, most animals were euthanized before advanced disease onset (Table 1). In summary, exploration and validation of such animal models is critical for the future exploration of therapeutic intervention strategies as is the development of non-human primate models for both HeV and NiV infection which has yet to be attempted in earnest.

## ANTIVIRAL THERAPEUTIC TARGETS

Although there are superb examples of effective antiviral drugs that target viral replication, such as the DNA chain-terminators used in the treatment of various herpes viruses and the numerous drugs that interfere with the replication of human immunodeficiency virus type 1 (HIV-1) (reviewed in [52]), for the vast majority of human viral pathogens there is a vast shortage of effective therapies. Importantly, to date, only one antiviral drug has been utilized during a henipavirus outbreak, ribavirin, which was first synthesized in1972 [53]. It is perhaps the best known alternative drug therapy, exhibiting antiviral activity against a variety of mostly RNA viruses. Ribavirin is an accepted treatment for several viral infections including RSV, arenaviral hemorrhagic-fevers such as Lassa virus and some members of the family *Bunyaviridae* (reviewed in [54]). Because of its global availability and broad antiviral properties it is often employed for the treatment of viral diseases under conditions where no other options are available except supportive care. During the NiV encephalitis outbreak in Malaysia, there was evidence that ribavirin exhibited some clinical benefit [54-56] with an apparent 36% reduction in mortality with little evidence of serious side effects. Thus, ribavirin appears to be one available antiviral option; however, with fatality approaching 75% in the most recent NiV outbreaks in Bangladesh and India, a more effective repertoire of antiviral agents is needed.

HeV and NiV are transmitted oranasally, spread systemically and infected individuals succumb to disease within 7 to 10 days. Clinical and experimental data demonstrate that multiple organ systems are affected and that the lungs and brain are major sites of virus replication. Viremia has been documented in both clinical and experimental infections; however, the kinetics and duration vary. For both viruses, acute and late-onset encephalitis has been documented clinically. The initial sites and duration of henipavirus replication upon infection are largely unknown, mainly due to a lack of extensive *in vivo* experiments. Consequently, the optimal target and time frame for drug intervention are not known. Theoretically, therapeutics that target the mucosa should reduce viral loads in the lung. Intravenous agents should decrease viral loads and reduce systemic spread. Although targeting the CNS may prove difficult, a significant reduction in viral loads peripherally may enable the host to generate a protective immune response.

To date, all novel henipavirus therapeutic agents that have been identified target and interfere with HeV and NiV virus entry and these candidates can be divided into two categories: those used for prevention of disease, including various vaccines, and those used for treatment post-exposure which includes antibodies, fusion inhibitors and soluble receptor molecules. The scope of this review will be limited to those agents tested using infectious HeV or NiV *in vitro* or *in vivo*.

## PREVENTION: VACCINE DEVELOPMENT

The use of safe and efficacious vaccines has been crucial to prevention strategies for several important viral pathogens in humans. Presently, there are some sixteen FDA-approved vaccines routinely used to prevent infection by virulent human pathogens and the majority comprise live-attenuated virus preparations (reviewed [57]). Although new reverse genetics systems have been developed for NiV [58], it is unlikely that a live-attenuated vaccine will be approved for any BSL4 virus including HeV and NiV. All successful human viral vaccines induce neutralizing antibodies that can cross-react with immunologically relevant strains of a virus [59]. Furthermore, neutralizing antibodies are the key vaccine-induced protective mechanisms in the case of some well known human paramyxoviruses such as mumps and MeV [60, 61]. For paramyxoviruses, it is the envelope glycoproteins that elicit the majority of neutralizing antibody in an infected host [3]. Indeed, for henipaviruses, it has been demonstrated that immunization of animals with recombinant NiV and HeV F or G glycoproteins, either as a live vaccinia virus vector or purified protein preparation can elicit potent cross-reactive virus neutralizing humoral responses in hamsters and rabbits, respectively [62, 63].

The first successful experimental henipavirus vaccine utilized recombinant vaccinia viruses that encoded NiV F or G glycoprotein. In these studies, vaccination protected against NiV challenge in golden hamsters and importantly, protection lasted five months post-challenge suggesting protection potentially from late-onset disease symptoms [62]. Although important for proof of principle, these recombinant viruses are not a viable human vaccine candidate due to the inherent risks of vaccinia virus vaccination. Poxvirus vectors have emerged as licensed veterinary vaccines as well as candidate vaccines for humans. Concerns about the safety of wild-type vaccinia virus have been addressed with the advent of attenuated poxviruses such as modified vaccinia virus Ankara (MVA), which is considered the vaccinia virus strain of choice for clinical investigation [64]. Although, recombinant MVA expressing HeV F or G has been generated, neither has been tested as a vaccine candidate (Bossart, K. and Broder, C.; unpublished data). Other poxvirus vectors shown to be attenuated in humans, such as the avipoxviruses, have also been explored as live vaccine vectors (reviewed in [65, 66]) and licensed feline, canine and equine canarypox virus-based vaccines are commercially available [67]. Recently, recombinant canarypox virus-based vaccines encoding NiV F or G successfully protected pigs from NiV challenge [68]. Interestingly, neutralizing antibodies and cell mediated immunity were examined and the study suggested that G-containing recombinant canarypox vaccines could elicit protective humoral and type 1 cell-mediated immune responses. Understanding and optimizing the immune response to henipaviruses represents a key aspect of human vaccine development. Importantly, this was the first report of recombinant canarypox virus vaccines being used in pigs and represents significant progress towards a veterinary vaccine for NiV. Furthermore, if evaluated successfully in an independent animal model, recombinant NiV F- and G-containing canarypox vaccines could potentially be used as human NiV vaccine candidates.

Recombinant subunit immunogens represent a viable avenue of vaccine development for the henipaviruses. These vaccines can be quickly implemented, are quite simple, and can be administered with no risk of infection. Recently, recombinant, soluble, oligomeric versions of the G glycoprotein of both HeV and NiV (sG) were generated as potential subunit vaccines [63]. Recombinant, purified sG preparations have been shown to be ideal immunogens that retain a number of important functional and antigenic properties including the ability to bind virus receptor, block virus infection, and elicit a robust polyclonal neutralizing antibody response in rabbits and mice [63]. The sG glycoprotein can also capture and isolate virus-specific neutralizing human monoclonal antibodies (mAbs) from naive recombinant libraries [69]. When HeV and NiV sG were used as subunit vaccines in a cat NiV challenge model, all animals were protected from disease [43]. The serum neutralization titers were on the order of 1:20,000 and represented the highest titers against henipaviruses obtained to date. The high neutralizing antibody titers and the absence of NiV genome in all vaccinated animals following challenge suggested that the sG subunit vaccines elicited sterilizing immunity. Unlike previous vaccines strategies, heterotypic protection was achieved where the attachment glycoprotein from HeV protected against challenge with NiV. If evaluated successfully in an additional animal model, these subunit vaccines may also represent viable human vaccine candidates. All of the potential vaccines discussed thus far are summarized in Table 2.

In order for a henipavirus vaccine to be considered for human use, not only does it need to be successful in two animal models, a natural route of infection should be used in protection studies, the mechanism and limits of protection should be elicited, and the highest standards should be used for reagents production to assure safety and success in human trials. Additionally, for HeV and NiV, we believe it would be ideal to have one vaccine that protects against both viruses. All of the above vaccine trials offer critical data towards these goals, but further refinement of reagents and protocols are necessary. Firstly, the selection of adjuvant will be important in human use. Secondly, a correlation between vaccine dose and protection will need to be established to ensure that vaccine preparations induce protective immune responses well above sub-optimal levels. Correlations of immunity will also be critical, including antibody subclasses such as IgA, IgG and IgM and their location (serum *vs* mucus membranes). Finally, it will be important to determine the role of cell-mediated type 1 immune responses in the strength and longevity of protective immunity.

To address these concerns, a new sG subunit vaccine trial has been initiated. HeV sG was chosen as the antigen because, based on previously established G-specific cross neutralization titers [43, 70], it can elicit the best cross-reactive henipavirus immune response (see Table 3). Varying doses of sG were employed in hopes of correlating protection with vaccine dose. Additionally, small CpG DNA molecules were used as the adjuvant for several reasons. Recent studies have demonstrated that CpG molecules can elicit mucosal and systemic type 1 cell-mediated immune responses regardless of the route of vaccine delivery. Equally important, several CpG molecules have been approved by the FDA for use in humans and numerous others are in clinical trials. All vaccinated animals were challenged oronasally with 50,000 TCID_50_ of a low passage NiV isolate. Preliminary pre-challenge results are summarized in Table 4. As expected, different doses of sG induced varying levels of serum neutralizing antibody, and titers were significantly lower to NiV as compared to HeV. Antigen-specific IgG and IgM were detected in all sera from all vaccinated animals, and sG-specific IgA was present in serum from one high dose animal. Interestingly, HeV sG-specific IgA was detected in the mucosal washes from all vaccinated animals. Post-challenge titers and protection from disease are not yet known, however, the data generated in the current vaccine trial will be valuable for future development of a human henipavirus vaccine.

## POST-EXPOSURE THERAPEUTICS

### Antibodies

While the neutralizing antibodies elicited by a vaccine can be highly effective, purified neutralizing antibodies administered passively to acutely infected individuals can be equally efficient. Passive antibody therapy is routinely used for prophylaxis against several important human pathogens including hepatitis B, varicella, RSV and rabies virus. With the advancement of mouse-human chimeric mAbs and the capability to ‘humanize’ murine mAbs, passive antibody therapy has become even more efficient. One such example includes a ‘humanized’ mAb to RSV F protein (Synagis®/Palivizumab), a more cost-effective and highly sucessful treatment compared to the original polyclonal product [71]. Passive therapy for NiV was first demonstrated using polyclonal hyper immune serum in hamsters [62]. Here, hamsters were administered anti-NiV F or anti-NiV G polyclonal sera prior to challenge and all animals survived subsequent NiV challenge. In more recent studies, murine mAbs directed against NiV F or G were evaluated in lethal NiV challenge experiments in hamsters [72]. When mAbs were administered prior to and shortly after challenge all animals were protected. However, when administered 24 hours post-challenge, only 50% of the animals survived. Together these data demonstrate that passive transfer is possible for NiV. Importantly, to be further developed as a post-exposure therapy for human use, efficacy of this treatment needs to be increased beyond a 24 hour window, and murine mAbs may need to be converted to more humanized molecules.

A major advancement in antibody technology has been the development of the phage display platform of combinatorial antibody libraries [73] as well as domain and chain shuffling methodologies to generate antibodies with new functional properties [74]. Phage libraries encode antibodies in the form of single-chain variable region fragments (scFvs) or Fab’ fragments. Human phage display platforms are the most extensively used and this technology has been complemented by innovative affinity maturation strategies to obtain high affinity reagents (reviewed [75]). These new techniques in human phage display platforms have been an enabling technology for the very rapid identification and isolation of specific mAbs, and have eliminated the time-consuming processes of immunization, hybridoma development and humanization techniques. Neutralizing human mAbs reactive to the G glycoprotein of HeV and NiV have been successfully identified, isolated, and characterized using these recombinant antibody techniques [69]. In particular, Fab’ fragment m102 had significant neutralizing activities against both henipaviruses and mapped to the receptor binding domain of HeV and NiV G [69, 70]. Subsequently, m102 was converted to a full length human IgG1 antibody, affinity maturated (m102.4) and evaluated in virus neutralization assays. The m102.4 human IgG antibody could completely neutralize both HeV and NiV at concentrations as low as 10 μg/ml (Zhu, Z. and Bossart, K.; unpublished data). In light of its potency *in vitro*, its mechanism of neutralization and the fact that is a fully human antibody, m102.4 could provide a valuable post-exposure or post-infection therapeutic for disease caused by HeV or NiV in humans. Pharmacological assays are currently under development and will be discussed below.

### Fusion Inhibitory Peptides

There have been considerable advances in mechanistic models of how several viral envelope glycoproteins function in driving the membrane fusion reaction (reviewed in [76-78]). A key component of many of these fusion glycoproteins is two α-helical domains referred to as heptad repeats (HR) that are involved in the formation of a trimer-of-hairpins structure [79, 80]. HR-1 is located proximal to the amino (N)-terminal fusion peptide and HR-2 precedes the transmembrane domain near the carboxyl (C)-terminus [79, 81-83]. For many viral fusion glycoproteins the N-terminal HR-1 forms an interior, trimeric coiled-coil surrounded by three anti-parallel helices formed from HR-2 and the formation of this structure mediates and provides the necessary energy to drive viral-host cell membrane merger (reviewed in [84]).

Over the past decade, targeting the viral membrane fusion process for antiviral drug development has received much attention, primarily lead by work on HIV-1 (reviewed in [85]). Importantly, the HIV-1 envelope derived peptide, enfuvirtide (Fuzeon™, formerly T-20), has been a clinical success [86, 87]. Enfuvirtide is a 36-amino acid peptide corresponding to a portion of the C-terminal HR-2 domain of the gp41 subunit of the envelope glycoprotein. Peptide sequences derived from F glycoprotein HR domains of several paramyxoviruses, including HeV and NiV have been shown to be potent inhibitors of fusion [88-93]. Moreover, HeV and NiV F glycoprotein HR domains have been shown to interact with each other and form the typical 6-helix coiled-coil bundles [94].

For the henipaviruses, second generation heptad-derived peptides were synthesized with chemical modifications of either the N- or C-terminus of the peptide by capping and/or “pegylation”, the coupling of polyethylene glycol (PEG) to proteins. Specific chemical modifications of the NiV F heptad-derived peptide were chosen, especially pegylation, to help improve plasma half-life and thus enhance therapeutic success. Pegylation is currently considered one of the most successful techniques to prolong the residence time of protein drugs in the bloodstream [95-98]. Like their predecessors, the new chemically modified heptad-derived peptides potently inhibited henipavirus infection *in vitro* in a dose-dependent fashion [99]. The plasma half-life of these peptides was recently evaluated in cats *in vivo* and was found to be unusually short (Crameri, G. and Broder, C., unpublished data), especially when compared to the half-life of Enfuvirtide in humans. As pure carnivores, cats have a unique metabolism designed to convert protein into sugar and this feature may have played a role in the rapid clearance of peptides from the bloodstream. For these reasons the peptide plasma half-life is currently being reevaluated in another animal species, ferrets. Once pharmacological studies have been completed and adequate peptide plasma half-life has been demonstrated, these peptides will be administered *in vivo* to examine their efficacy in treatment of ongoing NiV-mediated disease. It is hoped that, as was seen with enfuvirtide, the fusion inhibiting peptides might reduce viral load and or systemic spread *in vivo*.

### Soluble Receptor Molecules

**Fig. (2):**
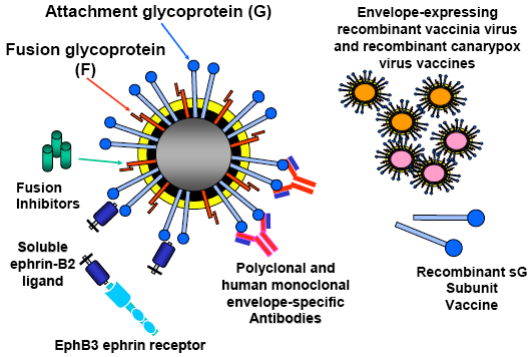
Henipavirus therapeutic agents evaluated *in vitro* and *in vivo*.

In addition to using virus-neutralizing human mAbs or peptides as a post-exposure therapeutic modality for HeV and NiV infection, a soluble virus receptor which binds G and prevents attachment of the virion to the host cell may also represent a feasible therapeutic strategy. It has been demonstrated that soluble ephrin-B2 ligand can block infectious henipavirus infection *in vitro* [27] and more recently, it has been demonstrated that soluble ephrin-B2 ligand could block both ephrin-B2 and ephrin-B3 ligand mediated henipavirus infections *in vitro* (Bossart, K. and Wang, L.; unpublished data). Although only recently identified as a functional receptor for the henipaviruses, the ligand ephrin-B2 and its cellular receptor EphB4, have attracted much attention due to their roles in cancer biology [100, 101]. Currently, soluble versions of both molecules are being explored as possible therapeutic drugs for human cancer. Specifically, blocking this receptor/ligand interaction inhibits the end stage of tumor blood vessel formation and causes direct growth inhibition of certain cancers [33, 102]. Interestingly, EphB4 has no inhibitory effects on ephrin-B3 ligand mediated henipavirus infection, only EphB3 a natural receptor for ephrin-B2 and ephrin-B3 ligands, was capable of blocking all henipavirus infections *in vitro* (Bossart, K. and Wang, L.; unpublished data). The potential passive therapeutic efficacy of soluble ephrin-B2 ligand or EphB3 protein in an animal model remains to be determined. However, their mechanism of action could be viewed as similar to that of a G glycoprotein-specific or receptor-specific antibody. A summary of all prevention and post-exposure henipavirus therapeutics is shown in Fig. (2).

### Pharmacology of Post-Exposure Therapeutics

**Fig. (3):**
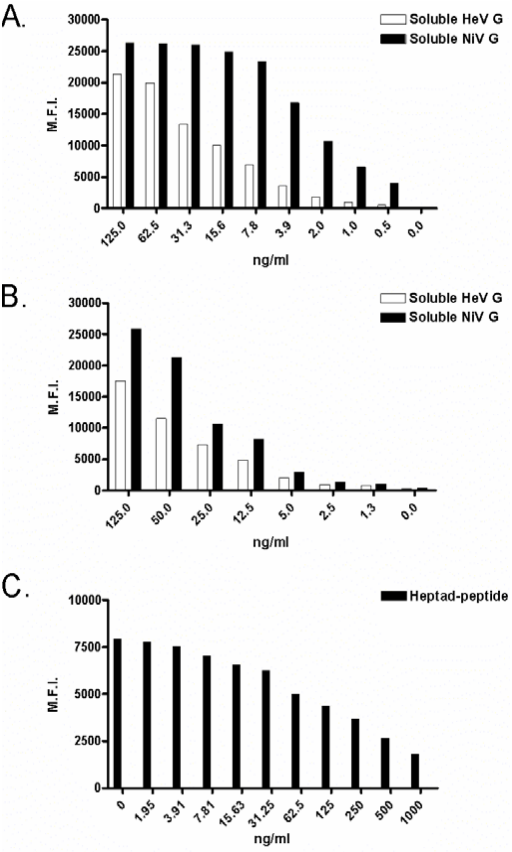
Standard curves using new pharmacological assays for various post-exposure therapeutic agents. For each graph, median fluorescent intensity (M.F.I.) is shown on the Y-axis and concentration is shown on the X-axis. For panels **A** and **B** binding to soluble HeV G- and soluble NiV G-coupled microspheres are indicated by white and black bars, respectively. For panel **C** binding of antibody to peptide-coupled microspheres in the presence and absence of free peptide is indicated by black bars. **A**: Binding of recombinant human m102.4 IgG1 monoclonal antibody; **B**: Binding of recombinant soluble ephrin-B2 ligand; **C**: Competition by soluble heptad-derived peptide.

A complete understanding of plasma half-life and toxicity are required for the development of any human use therapeutic. The pharmacological properties of virus-specific human mAbs and heptad-derived peptides are currently being evaluated *in vivo*, and we believe that soluble ephrin-B2 ligand and EphB3 studies will commence in the not too distant future. To aid drug measurement *ex vivo*, new assays that exhibit extraordinary sensitivity have been developed. Differential henipavirus serology and neutralization assays using Bio-plex array systems were recently described [70]. These assays used HeV and NiV sG-coupled fluorescently-labeled microspheres, test sera or soluble receptors and phycoerythrin-labeled detection molecules and binding results were reported as median fluorescent intensities. Although developed to detect host antibodies that interfered with receptor binding, these assays were easily adapted for measuring therapeutics that bound the attachment glycoproteins of HeV and NiV. The sG coupled-microspheres have been used to detect ephrin-B2 ligand and G-specific human mAbs, with approximate sensitivities of 5 ng/ml and 250 pg/ml; respectively (Bossart, K. unpublished data). Clearly, these assays will prove valuable for detecting soluble ephrin-B2 ligand in sera and are already being used to measure G-specific human mAbs in sera during plasma half-life experiments. Finally, another microsphere-based assay capable of measuring F-specific heptad-derived peptides in sera has been developed. In this assay, microspheres coated with heptad-derived peptide are detected using a peptide-specific polyclonal antibody and anti-rabbit-phycoerythrin molecules and results are reported as median fluorescent intensities. When test sera are added, free peptide competes for antibody binding and thus decreased fluorescent signals are detected. Similar to the other microsphere assays, this new peptide assay has an approximate sensitivity of 5 ng/ml Examples of standard curves for all assays are shown in Fig. (3). The development of such assays will enable accurate measurement of each potential therapeutic agent *ex vivo* and set the stage for evaluating the efficacy of each in henipavirus animal challenge models.

## CONCLUDING REMARKS

The increased understanding of henipavirus pathogenesis has lead to the development of suitable animal models of disease and potential therapeutics. The successful vaccine candidates have been trialed in one animal model but still need to be evaluated in an independent animal model. Additionally, as discussed, while providing important proof of concept data, further understanding of protection mechanisms and refinement of reagents will be critical if any vaccine candidate is to progress further. Most of the post-exposure therapeutic agents have only been tested *in vitro* and will require *in vivo* half-life studies and disease prevention efficacy studies in at least two animal models. The complexities of such studies include optimizing drug delivery *in vivo* and site-specific targeting of post-exposure antivirals. Although many avenues of henipavirus therapeutic research have been successful, it will most likely take at least five to ten years before any therapeutic agent undergoes all necessary testing, gains FDA approval and is used routinely during an outbreak.

An understanding of the molecular biology of HeV and NiV has expanded rapidly in recent years. Although this review has been limited to those agents tested against infectious virus, numerous *in vitro* cell culture systems have been developed for examining various henipavirus gene-specific functions. Consequently, protein-based mechanisms, such as attachment and fusion, F cleavage, interferon interference and virus particle formation have been further elicited [103]. Importantly, these processes all represent targets for new antiviral drug development and both high throughput screening and specific inhibition assays are well underway. The construction of a NiV reverse genetics system [58], although unlikely to be used in vaccine trials, will significantly aid gene knock out studies using infectious clones and new therapeutic targets will most likely be identified. New therapeutic gene silencing techniques, such as small interfering RNA molecules, are also being developed for NiV. Characterized genes are the current targets; however, new genome regions identified using reverse genetics also represent potential sequence targets. The vast expansion of henipavirus molecular biology has underpinned new drug discovery; however, virus neutralization assays, drug characterization *in vivo* and efficacy studies still represent the most challenging phases of drug development for HeV and NiV, especially under BSL-4 restrictions.

## Figures and Tables

**Table 1:** Small Animal Models for Henipavirus Infection

	Challenge Dose			Pathology			Ref.
	Intra-Peritoneal	Subcutaneous	Intranasal-Oral-Oronasal	General	Lung	Brain	
**Guinea pig HeV**		5,000 TCID_50_ 50,000 TCID_50_		Weakness, lethargy, abortion, head tilt. Death or euthanasia 6-15 days PI. Vascular degeneration in multiple organs. Immuno-staining of blood vessels.	Fibrinoid degeneration of blood vessels, syncytia of endothelium and alveolar walls	microscopical lesions of encephalitis in 8/15 guineapigs subcutaneously inoculated with 30,000-50,000 TCID_50_	[36-38]
**Guinea pig NiV**	50,000 TCID_50_			Mild behavioral changes, ataxia. Euthanasia 7 – 10 DPI. Infection in 3/8 animals. Vasculitis, fibrinoid necrosis of vessels in multiple organs, oophoritis, endometrial necrosis. Antigen in endothelium, syncytia, blood vessel walls, myometrium and endometrium.	Lung lesions not observed	Encephalitis not observed	[39]
**Golden Hamster NiV**	100 – 10,000 pfu		1000 – 1,000,000 pfu	Tremors, paralysis, lethargy, breathing difficulty. Death 9 – 29 DPI. Vascular fibrinoid necrosis and inflammation in multiple organs. Antigen and genome in endothelial cells, syncytia and tunica media of blood vessels.	Paranchymal inflammation, vasculitis **	Vasculitis. Mild inflammation of parenchyma and meninges. Antigen, genome and inclusion bodies in neurons **	[40]
**Cat HeV**		5,000 TCID_50_	50,000 TCID_50_	Death or euthanasia 6-7 DPI. Vascular lesions and paranchymal degeneration in gastrointestinal tract and lymphoid organs.	Interstitial pneumonia, vascular necrosis, endothelial syncytia. **	Encephalitis not reported	[36, 41]
**Cat NiV**		500 TCID_50_	50,000 TCID_50_	Euthanasia 7-9 DPI after short febrile illness and increase in respiratory rate; clinical resolution in one cat. Necrosis in spleen, inflammation of bladder, necrotizing lymphadenitis.	Focal necrotizing alveolitis with syncytia, bronchiolitis, fibrinoid necrosis of blood vessel walls, endothelial syncytia. Antigen in affected bronchial and alveolar epithelium, syncytia and blood vessels. **	Meningitis, meningeal vasculitis with endothelial syncytia; distinct encephalitis has not been identified	[42, 43]
**Ferret Ni**V*			500-50,000 TCID_50_	Fever, depression and weakness. Euthanasia 6 – 10 DPI following 1 – 2 days fever. Vascular fibrinoid necrosis in multiple organs, necrotising alveolitis, syncytia of endothelium and alveolar epithelium, necrotizing lymphadenitis. Antigen in blood vessel walls and syncytia.	Vascular fibrinoid necrosis, necrotising alveolitis, syncytia of alveolar epithelium. Antigen in blood vessel walls and syncytia. **	Not yet determined	Unpublished (Bossart, K, Bingham, J, and Middleton, D)

* Preliminary data: pathology has not yet been completed;

** Similar to human pathology.

**Table 2:** Effective Henipavirus Vaccines

	Immunization Schedule	Adjuvant	Challenge: Dose and Route	Immune Correlates of Survival
**Vaccinia viruses encoding NiV F or G (hamsters)**	2 subcutaneous doses (10^7^ pfu per dose) 1 month apart	None	3 months post immunization, 1000 pfu NiV intraperitoneally	Viral load Antibodies (ELISA and SNT)
**Canarypox viruses encoding NiV F or G (pigs)**	2 intramuscular doses (10^8^ pfu per dose) 14 days apart	None	29 days post immunization, 2.5 x 10^5^ pfu NiV intranasally	Viral load Cytokine production Antibodies (ELISA, IFA and SNT)
**Recombinant HeV or NiV soluble G (cats)**	3 subcutaneous doses (100 µg per dose) 14 days apart	CSIRO triple adjuvant	11 weeks post immunization, 500 TCID_50_ NiV subcutaneously	Viral load Antibodies (SNT)

**Table 3:** Examples of Heterotypic Henipavirus Serum Titers

Sera from Infected Individuals	Binding to NiV G	Binding to HeV G	Sera from sG Vaccinated Cats	SNT Titer NiV	SNT Titer HeV
HeV human	1:16000	1:8000	HeV sG cat 1	1:20480	1:20480
HeV horse	1:16000	1:32000	HeV sG cat 2	1:20480	1:20480
NiV human	1:32000	1:2000	NiV sG cat 3	1:20480	1:1280
NiV pig	1:8000	neg	NiV sG cat 4	1:20480	1:2560

SNT = serum neutralization test.

**Table 4:** Antibody Profiles of sG Vaccinated Cats

	HeV SNT Titer	NiV SNT Titer	sG-specific Serum IgG	sG-specific Serum IgM	sG-specific Serum IgA	HeV sG-Specific Mucosal IgA
Control	1:32	< 1:2	-	-	-	-
Control	1:16	< 1:2	-	-	-	-
High dose	1:4096	1:512	+	+	+	+
High dose	1:2048	1:256	+	+	-	+
Medium dose	1:4096	1:512	+	+	-	+
Medium dose	1:4096	1:256	+	+	-	+
Low dose	1:1024	1:128	+	+	-	+
Low dose	1:1024	1:32	+	+	-	+

SNT = serum neutralization test; sG-specific = antibodies detected to both HeV and NiV sG.

## References

[1] Halpin K, Young PL, Field HE, Mackenzie JS (2000). Isolation of Hendra virus from pteropid bats: a natural reservoir of Hendra virus. J Gen Virol.

[2] Chua KB, Lek Koh C, Hooi PS (2002). Isolation of Nipah virus from Malaysian Island flying-foxes. Microbes Infect.

[3] Lamb RA, Parks GD, Knipe DM, Howley PM (2007). *Paramyxoviridae*: The viruses and their replication. Fields Virology.

[4] Wang L, Harcourt BH, Yu M (2001). Molecular biology of Hendra and Nipah viruses. Microbes Infect.

[5] Eaton BT, Broder CC, Middleton D, Wang LF (2006). Hendra and Nipah viruses: different and dangerous. Nat Rev Microbiol.

[6] Westbury HA (2000). Hendra virus disease in horses. Rev Sci Tech.

[7] Hanna JN, McBride WJ, Brookes DL (2006). Hendra virus infection in a veterinarian. Med J Aust.

[8] Anonymous. Hendra virus, equine - Australia (NSW): susp. International Society for Infectious Diseases 2006; Report No.: 20061109.3222.

[9] Chua KB (2003). Nipah virus outbreak in Malaysia. J Clin Virol.

[10] Tan CT, Wong KT (2003). Nipah encephalitis outbreak in Malaysia. Ann Acad Med Singapore.

[11] Hsu VP, Hossain MJ, Parashar UD (2004). Nipah Virus Encephalitis Reemergence, Bangladesh. Emerg Infect Dis.

[12] Harit AK, Ichhpujani RL, Gupta S (2006). Nipah/Hendra virus outbreak in Siliguri, West Bengal, India in 2001. Indian J Med Res.

[13] (2005). Anonymous. Emerging Infections update: November 2004 to January 2005. Communicable Disease Report Weekly (CDR Weekly).

[14] (2004). Anonymous. Nipah encephalitis outbreak over wide area of western Bangladesh, 2004. Health and Science Bulletin (ICDDR,B).

[15] Anonymous. Nipah virus, fatal - India (West Bengal) (02). International Society for Infectious Diseases 2007; Report No.: 20070511. 1514.

[16] Gurley E, Montgomery JM, Hossain MJ, Bell M, Azad AK, Islam MR (2007). Person-to-person transmission of Nipah virus in a Bangladeshi community. Emerg Infect Dis.

[17] Luby SP, Rahman M, Hossain MJ (2006). Foodborne transmission of Nipah virus, Bangladesh. Emerg Infect Dis.

[18] Hooper P, Zaki S, Daniels P, Middleton D (2001). Comparative pathology of the diseases caused by Hendra and Nipah viruses. Microbes Infect.

[19] Lam SK, Chua KB (2002). Nipah virus encephalitis outbreak in Malaysia. Clin Infect Dis.

[20] Wong KT, Shieh WJ, Kumar S (2002). Nipah virus infection: pathology and pathogenesis of an emerging paramyxoviral zoonosis. Am J Pathol.

[21] Dorig RE, Marcil A, Chopra A, Richardson CD (1993). The human CD46 molecule is a receptor for measles virus (Edmonston strain). Cell.

[22] Naniche D, Varior-Krishnan G, Cervoni F (1993). Human membrane cofactor protein (CD46) acts as a cellular receptor for measles virus. J Virol.

[23] Nussbaum O, Broder CC, Moss B, Stern LB, Rozenblatt S, Berger EA (1995). Functional and structural interactions between measles virus hemagglutinin and CD46. J Virol.

[24] Tatsuo H, Ono N, Tanaka K, Yanagi Y (2000). SLAM (CDw150) is a cellular receptor for measles virus. Nature.

[25] Tatsuo H, Ono N, Yanagi Y (2001). Morbilliviruses use signaling lymphocyte activation molecules (CD150) as cellular receptors. J Virol.

[26] Baron MD (2005). Wild-type Rinderpest virus uses SLAM (CD150) as its receptor. J Gen Virol.

[27] Bonaparte MI, Dimitrov AS, Bossart KN (2005). Ephrin-B2 ligand is a functional receptor for Hendra virus and Nipah virus. Proc Natl Acad Sci USA.

[28] Negrete OA, Levroney EL, Aguilar HC (2005). EphrinB2 is the entry receptor for Nipah virus, an emergent deadly paramyxovirus. Nature.

[29] Negrete OA, Wolf MC, Aguilar HC (2006). Two key sesidues in EphrinB3 are critical for its use as an alternative receptor for Nipah virus. PLoS Pathog.

[30] Poliakov A, Cotrina M, Wilkinson DG (2004). Diverse roles of Eph receptors and ephrins in the regulation of cell migration and tissue assembly. Dev Cell.

[31] Drescher U (2002). Eph family functions from an evolutionary perspective. Curr Opin Genet Dev.

[32] Palmer A, Klein R (2003). Multiple roles of ephrins in morphogenesis, neuronal networking, and brain function. Genes Dev.

[33] Wang HU, Chen ZF, Anderson DJ (1998). Molecular distinction and angiogenic interaction between embryonic arteries and veins revealed by ephrin-B2 and its receptor Eph-B4. Cell.

[34] Adams RH, Wilkinson GA, Weiss C (1999). Roles of ephrinB ligands and EphB receptors in cardiovascular development: demarcation of arterial/venous domains, vascular morphogenesis, and sprouting angiogenesis. Genes Dev.

[35] Bossart KN, Broder CC, Pohlmann S, Simmons G (2007). Paramyxovirus entry. Viral Entry into Host Cells.

[36] Hooper PT, Westbury HA, Russell GM (1997). The lesions of experimental equine morbillivirus disease in cats and guinea pigs. Vet Pathol.

[37] Williamson MM, Hooper PT, Selleck PW, Westbury HA, Slocombe RF (2000). Experimental hendra virus infectionin pregnant guinea-pigs and fruit Bats (Pteropus poliocephalus). J Comp Pathol.

[38] Williamson MM, Hooper PT, Selleck PW, Westbury HA, Slocombe RF (2001). A guinea-pig model of Hendra virus encephalitis. J Comp Pathol.

[39] Middleton DJ, Morrissy CJ, van der Heide BM (2007). Experimental Nipah Virus Infection in Pteropid Bats (Pteropus poliocephalus). J Comp Pathol.

[40] Wong KT, Grosjean I, Brisson C (2003). A golden hamster model for human acute Nipah virus infection. Am J Pathol.

[41] Westbury HA, Hooper PT, Brouwer SL, Selleck PW (1996). Susceptibility of cats to equine morbillivirus. Aust Vet J.

[42] Middleton DJ, Westbury HA, Morrissy CJ (2002). Experimental Nipah virus infection in pigs and cats. J Comp Pathol.

[43] Mungall BA, Middleton D, Crameri G (2006). Feline model of acute Nipah virus infection and protection with a soluble glycoprotein-based subunit vaccine. J Virol.

[44] Hooper PT, Ketterer PJ, Hyatt AD, Russell GM (1997). Lesions of experimental equine morbillivirus pneumonia in horses. Vet Pathol.

[45] Yob JM, Field H, Rashdi AM (2001). Nipah virus infection in bats (order Chiroptera) in peninsular Malaysia. Emerg Infect Dis.

[46] Westbury HA, Hooper PT, Selleck PW, Murray PK (1995). Equine morbillivirus pneumonia: susceptibility of laboratory animals to the virus. Aust Vet J.

[47] Zitzow LA, Rowe T, Morken T, Shieh WJ, Zaki S, Katz JM (2002). Pathogenesis of avian influenza A (H5N1) viruses in ferrets. J Virol.

[48] Martina BE, Haagmans BL, Kuiken T (2003). Virology: SARS virus infection of cats and ferrets. Nature.

[49] Svitek N, von Messling V (2007). Early cytokine mRNA expression profiles predict Morbillivirus disease outcome in ferrets. Virology.

[50] Ball RS (2006). Issues to consider for preparing ferrets as research subjects in the laboratory. Ilar J.

[51] Gad S (2000). Pigs and ferrets as models in toxicology and biological safety assessment. Int J Toxicol.

[52] Coen DM, Richman DD, Knipe DM, Howley PM (2007). Antiviral agents. Fields Virology.

[53] Sidwell RW, Huffman JH, Khare GP, Allen LB, Witkowski JT, Robins RK (1972). Broad-spectrum antiviral activity of Virazole: 1-beta-D-ribofuranosyl-1,2,4-triazole-3-carboxamide. Science.

[54] Snell NJ (2001). Ribavirin--current status of a broad spectrum antiviral agent. Expert Opin Pharmacother.

[55] Chong HT, Kamarulzaman A, Tan CT (2001). Treatment of acute Nipah encephalitis with ribavirin. Ann Neurol.

[56] Snell NJ (2004). Ribavirin therapy for nipah virus infection. J Virol.

[57] Graham BS, Crowe JE, Knipe DM, Howley PM (2007). Immunization Against Viral Diseases. Fields Virology.

[58] Yoneda M, Guillaume V, Ikeda F (2006). Establishment of a Nipah virus rescue system. Proc Natl Acad Sci USA.

[59] Quinnan GV, Galasso G, Whitley R, Merigan TC (1997). Immunization against viral diseases. Antiviral Agents and Human Viral Disease.

[60] Pantaleo G, Koup RA (2004). Correlates of immune protection in HIV-1 infection: what we know, what we don’t know, what we should know. Nat Med.

[61] Griffin DE (1995). Immune responses during measles virus infection. Curr Top Microbiol Immunol.

[62] Guillaume V, Contamin H, Loth P (2004). Nipah virus: vaccination and passive protection studies in a hamster model. J Virol.

[63] Bossart KN, Crameri G, Dimitrov AS (2005). Receptor binding, fusion inhibition and induction of cross-reactive neutralizing antibodies by a soluble G glycoprotein of Hendra virus. J Virol.

[64] Sutter G, Staib C (2003). Vaccinia vectors as candidate vaccines: the development of modified vaccinia virus Ankara for antigen delivery. Curr Drug Targets Infect Disord.

[65] Franchini G, Gurunathan S, Baglyos L, Plotkin S, Tartaglia J (2004). Poxvirus-based vaccine candidates for HIV: two decades of experience with special emphasis on canarypox vectors. Expert Rev Vaccines.

[66] Broder CC, Earl PL (1999). Recombinant vaccinia viruses. Design, generation, and isolation. Mol Biotechnol.

[67] Poulet H, Brunet S, Boularand C (2003). Efficacy of a canarypox virus-vectored vaccine against feline leukaemia. Vet Rec.

[68] Weingartl HM, Berhane Y, Caswell JL (2006). Recombinant nipah virus vaccines protect pigs against challenge. J Virol.

[69] Zhu Z, Dimitrov AS, Bossart KN (2006). Potent neutralization of Hendra and Nipah viruses by human monoclonal antibodies. J Virol.

[70] Bossart KN, McEachern JA, Hickey AC (2007). Neutralization assays for differential henipavirus serology using Bio-Plex Protein Array Systems. J Virol Methods.

[71] Zeitlin L, Cone RA, Moench TR, Whaley KJ (2000). Preventing infectious disease with passive immunization. Microbes Infect.

[72] Guillaume V, Contamin H, Loth P (2006). Antibody prophylaxis and therapy against Nipah virus infection in hamsters. J Virol.

[73] Rader C, Barbas CF (1997). Phage display of combinatorial antibody libraries. Curr Opin Biotechnol.

[74] Hayden MS, Gilliland LK, Ledbetter JA (1997). Antibody engineering. Curr Opin Immunol.

[75] Hudson PJ, Souriau C (2001). Recombinant antibodies for cancer diagnosis and therapy. Expert Opin Biol Ther.

[76] Weissenhorn W, Dessen A, Calder LJ, Harrison SC, Skehel JJ, Wiley DC (1999). Structural basis for membrane fusion by enveloped viruses. Mol Membr Biol.

[77] Chan DC, Kim PS (1998). HIV entry and its inhibition. Cell.

[78] Skehel JJ, Wiley DC (1998). Coiled coils in both intracellular vesicle and viral membrane fusion. Cell.

[79] Singh M, Berger B, Kim PS (1999). LearnCoil-VMF: computational evidence for coiled-coil-like motifs in many viral membrane-fusion proteins. J Mol Biol.

[80] Hughson FM (1997). Enveloped viruses: a common mode of membrane fusion?. Curr Biol.

[81] Xu Y, Gao S, Cole DK (2004). Basis for fusion inhibition by peptides: analysis of the heptad repeat regions of the fusion proteins from Nipah and Hendra viruses, newly emergent zoonotic paramyxoviruses. Biochem Biophys Res Commun.

[82] Bossart KN, Wang LF, Eaton BT, Broder CC (2001). Functional expression and membrane fusion tropism of the envelope glycoproteins of Hendra virus. Virology.

[83] Chambers P, Pringle CR, Easton AJ (1990). Heptad repeat sequences are located adjacent to hydrophobic regions in several types of virus fusion glycoproteins. J Gen Virol.

[84] Earp LJ, Delos SE, Park HE, White JM (2005). The many mechanisms of viral membrane fusion proteins. Curr Top Microbiol Immunol.

[85] Weiss CD (2003). HIV-1 gp41: mediator of fusion and target for inhibition. AIDS Rev.

[86] Kilby JM, Hopkins S, Venetta TM (1998). Potent suppression of HIV-1 replication in humans by T-20, a peptide inhibitor of gp41-mediated virus entry. Nat Med.

[87] Kilby JM, Lalezari JP, Eron JJ (2002). The safety, plasma pharmacokinetics, and antiviral activity of subcutaneous enfuvirtide (T-20), a peptide inhibitor of gp41-mediated virus fusion, in HIV-infected adults. AIDS Res Hum Retroviruses.

[88] Lambert DM, Barney S, Lambert AL (1996). Peptides from conserved regions of paramyxovirus fusion (F) proteins are potent inhibitors of viral fusion. Proc Natl Acad Sci USA.

[89] Joshi SB, Dutch RE, Lamb RA (1998). A core trimer of the paramyxovirus fusion protein: parallels to influenza virus hemagglutinin and HIV-1 gp41. Virology.

[90] Wild TF, Buckland R (1997). Inhibition of measles virus infection and fusion with peptides corresponding to the leucine zipper region of the fusion protein. J Gen Virol.

[91] Young JK, Li D, Abramowitz MC, Morrison TG (1999). Interaction of peptides with sequences from the Newcastle disease virus fusion protein heptad repeat regions. J Virol.

[92] Rapaport D, Ovadia M, Shai Y (1995). A synthetic peptide corresponding to a conserved heptad repeat domain is a potent inhibitor of Sendai virus-cell fusion: an emerging similarity with functional domains of other viruses. EMBO J.

[93] Bossart KN, Wang LF, Flora MN (2002). Membrane fusion tropism and heterotypic functional activities of the Nipah virus and Hendra virus envelope glycoproteins. J Virol.

[94] Xu Y, Lou Z, Liu Y (2004). Crystallization and preliminary crystallographic analysis of the fusion core from two new zoonotic paramyxoviruses, Nipah virus and Hendra virus. Acta Crystallogr D Biol Crystallogr.

[95] Harris JM, Martin NE, Modi M (2001). Pegylation: a novel process for modifying pharmacokinetics. Clin Pharmacokinet.

[96] Delgado C, Pedley RB, Herraez A (1996). Enhanced tumour specificity of an anti-carcinoembrionic antigen Fab’ fragment by poly(ethylene glycol) (PEG) modification. Br J Cancer.

[97] Delgado C, Malik F, Selisko B, Fisher D, Francis GE (1994). Quantitative analysis of polyethylene glycol (PEG) in PEG-modified proteins/cytokines by aqueous two-phase systems. J Biochem Biophys Methods.

[98] Caliceti P, Veronese FM (2003). Pharmacokinetic and biodistribution properties of poly(ethylene glycol)-protein conjugates. Adv Drug Deliv Rev.

[99] Bossart KN, Mungall BA, Crameri G, Wang LF, Eaton BT, Broder CC (2005). Inhibition of Henipavirus fusion and infection by heptad-derived peptides of the Nipah virus fusion glycoprotein. Virol J.

[100] Masood R, Xia G, Smith DL (2005). Ephrin B2 expression in Kaposi sarcoma is induced by human herpesvirus type 8: phenotype switch from venous to arterial endothelium. Blood.

[101] Martiny-Baron G, Korff T, Schaffner F (2004). Inhibition of tumor growth and angiogenesis by soluble EphB4. Neoplasia.

[102] Surawska H, Ma PC, Salgia R (2004). The role of ephrins and Eph receptors in cancer. Cytokine Growth Factor Rev.

[103] Eaton BT, Mackenzie JS, Wang LF, Knipe DM, Howley PM (2007). Henipaviruses. Fields Virology.

